# Population-Based Study of Acute Respiratory Infections in Children, Greenland

**DOI:** 10.3201/eid0806.010321

**Published:** 2002-06

**Authors:** Anders Koch, Per Sørensen, Preben Homøe, Kåre Mølbak, Freddy Karup Pedersen, Tine Mortensen, Hanne Elberling, Anne Mette Eriksen, Ove Rosing Olsen, Mads Melbye

**Affiliations:** * Statens Serum Institut, Copenhagen, Denmark; † National University Hospital, Copenhagen, Denmark; ‡ Sisimiut Health Center, Sisimiut, Greenland

## Abstract

Acute respiratory infections (ARI) are frequent in Inuit children, in terms of incidence and severity. A cohort of 294 children <2 years of age was formed in Sisimiut, a community on the west coast of Greenland, and followed from 1996 to 1998. Data on ARI were collected during weekly visits at home and child-care centers; visits to the community health center were also recorded. The cohort had respiratory symptoms on 41.6% and fever on 4.9% of surveyed days. The incidence of upper and lower respiratory tract infections was 1.6 episodes and 0.9 episodes per 100 days at risk, respectively. Up to 65% of the episodes of ARI caused activity restriction; 40% led to contact with the health center. Compared with studies from other parts of the world, the incidence of ARI appears to be high in Inuit children.

In children of the Inuit, the aboriginal Eskimo population of the Arctic, acute respiratory infections (ARI) are frequent, measured in terms of incidence and severity. Infant death and disease from ARI are higher than in Denmark, United States, and Canada (1–3); many Inuit children have severe lower respiratory tract infections (LRI) early in life (4). Childhood otitis media, with an occurrence rate among the highest in the world (5–7), is characterized by early age at onset and a high chronicity (6–9). The causes of the high rates of otitis media are largely unknown, but nasopharyngeal carriage of potentially pathogenic bacteria and viruses in Greenlandic children in combination with frequent upper respiratory tract infections (URI) may be important (10).

To determine the incidence of ARI on the basis of population, we established a cohort of children <2 years of age in Sisimiut, a community on the west coast of Greenland. The goals of this study were to determine the epidemiology of acute respiratory tract infections in children on a prospective and longitudinal basis and to identify risk factors for such disease.

## Materials and Methods

### Study Area

Sisimiut is the second largest town in Greenland (pop. 5,117, January 1996). Of these inhabitants, 88% were born in Greenland and 12% outside Greenland, primarily in Denmark (11). In our study population, each household had a median of three rooms (interquartile range [IQR] three to four) and four persons (IQR four to five). Eighty percent of children lived in nuclear families with two parents, and 70% attended child-care centers during the study period.

All health services in Sisimiut, except for a dental clinic, are located at the community health center, which serves as general practice facility, birth clinic, and regular hospital. All births in the town take place at the center. All health services in Greenland, including prescribed medication, are free of charge.

### Study Population

The cohort consisted of all children <2 years living in Sisimiut from April 1, 1996, to June 1, 1998, including all children born there and all children who moved there during that period. To ensure that we included all Sisimiut children in the cohort, we obtained information on inhabitants in Sisimiut at regular intervals from the local authorities and from the Civil Registration System of Greenland (12), in which all citizens of Greenland are registered. Parents of children eligible for inclusion in the study were contacted by letter or in person. Using a standardized interview, trained project staff visited the home to obtain written informed consent and collect background information. Families who declined participation were asked their reasons by open-ended questions. Because visiting nurses see newborns in Sisimiut for the first 5 weeks, most children were enrolled after 6 weeks, although 12 children were, for the mothers’ convenience, enrolled before that time. The Commission for Scientific Research in Greenland, the scientific ethical board for research, approved the study.

### Illness Surveillance

From July 30, 1996, to August 13, 1998, children were monitored through weekly visits in their homes or child-care centers. Children absent from child-care centers at the scheduled time were visited at home. At all visits a standardized medical history based on the presence of respiratory symptoms (nasal secretion, cough, earache, ear discharge, hoarseness, sore throat, rapid or difficult breathing, or chest indrawing), fever, diarrhea (loose or watery stools >3 times a day), and signs of general malaise since the last information was obtained from the parents. Symptoms were recalled for the prior 1 to 2 weeks, and the exact days that symptoms occurred were recorded. If the parents reported one or more respiratory symptoms for the preceding week, a clinical examination focusing on the respiratory system was done including nonpneumatic otoscopy and tympanometry (MicroTymp2 tympanometers, Welch-Allyn, NY). The presence of diarrhea or fever alone did not prompt a clinical examination.

At the end of the study period, the children’s inpatient and outpatient charts kept at the community health center were reviewed, and doctors’ diagnoses or reporting of characteristic clinical signs of URI (common cold, pharyngitis, tonsillitis, or otitis media) and LRI (croup, bronchitis, bronchiolitis, or pneumonia) were noted.

### Case Definitions

An ARI episode, first reported as respiratory symptoms by the parents, was characterized as a URI or LRI, if confirmed by clinical examinations made by medical students as part of the study or by local doctors at the health center. The medical students used a modified set of signs proposed by the Board on Science and Technology for International Development (BOSTID) studies (13). A diagnosis of URI included one or more of the following clinical signs: purulent nasal discharge; cough; a red, bulging tympanic membrane with loss of normal landmarks and abnormal tympanometry; purulent ear discharge; and pharyngo-tonsillar erythema or exudate without signs of LRI. A diagnosis of LRI was made if one or more of the following clinical signs was present: respiratory rate >50/min and labored breathing or cough; rales; stridor; wheezing; cyanosis; or chest indrawing. During data analysis the presence of clear nasal discharge as the only finding was omitted from clinical definitions. Health center visits for rhinitis, pharyngitis, tonsillitis, or otitis media were regarded as URI, and visits for croup, bronchitis, bronchiolitis, or pneumonia were included as LRI. Episodes of respiratory infections, during which more than one clinical examination was conducted, were classified as LRI if one of the examinations met the criteria for LRI.

The minimum interval between two episodes of respiratory symptoms was considered to be 7 consecutive days. We defined prevalence as the number of days with a given symptom reported positive, divided by total number of days of observation, and we calculated incidence as the number of new episodes divided by person time at risk (14). Time at risk was defined as the number of days with no recorded symptoms including the first day of any episode, but excluding the 7 consecutive days without symptoms following an episode. The number of days of a given episode was considered to be its duration. If the last day of an episode was not followed by 7 consecutive days with no symptoms recorded (for example, in the case of missing information), this time period was truncated at the last day with recorded symptoms.

Severity was assessed by using the following measures: duration of illness, activity restriction, and visit or admission to health center. We used parental reporting to note activity restriction, including the child’s general condition, confinement to bed, absence from the child-care center, change in sleeping and eating patterns, and presence of fever.

### Statistical Methods

Where appropriate, chi-square test and Fisher’s exact test were used to test differences in distribution. Assuming a Poisson distribution for the number of episodes, we estimated 95% confidence intervals (CI); likelihood-ratio tests were used to test differences in incidence with respect to sex, age, and calendar period. The distribution of episode duration was estimated by using the nonparametric Kaplan-Meier estimator, thereby taking into account that some episodes were truncated. All statistical analyses were performed by using SAS v. 6.12 (SAS Institute Inc., Cary, NC). The GENMOD procedure was used for the Poisson regression analysis and the LIFETEST procedure to calculate Kaplan-Meier estimates (15, 16).

## Results

### Study Population

Of 356 children eligible for study, consent was obtained for 312 (87.6%), and 44 (12.4%) refused. For those who refused, reasons given were lack of time, various non-disease-related reasons, and medical issues not related to ARI; 15 gave no reason. Of the group of 312 children, 17 children passed their second birthday before the initial visit was made and were not included. One child was excluded during the study period for laryngomalacia, leaving 294 children as the study population (Table 1). Of the study population, 242 (82.3%) children participated for the scheduled period; 52 (17.7%) withdrew. Of those who withdrew, 37 (71.2%) moved out of Sisimiut, 14 (26.9%) declined to continue, and 1 died of aspiration pneumonia caused by febrile convulsions. Of those who declined to continue, none gave disease-related causes for withdrawal. The participation rate among Greenlandic children was higher than among Danish children, and children resident in Sisimiut at the beginning of the study were more likely to participate than children born in or moving into Sisimiut after the study began (Table 1).

### Illness Surveillance

The median age of the children at enrollment was 142 days; the median age at first day of illness information in the monitoring period was 174 days. Information on respiratory symptoms was obtained for a median of 256 days (Table 1).

In total, 11,081 interviews with the parents or guardians were attempted during the monitoring period; in 1,638 (14.8%) of these attempts, no contact was made. Of the 9,443 successful interviews, 34.2% were made in the children’s homes, 34.8% in child-care centers, and 14.1% by telephone. In 16.9% of the interviews, the place of interview was not noted.

### Respiratory Symptoms, Fever, and Diarrhea

**Figure 1:**
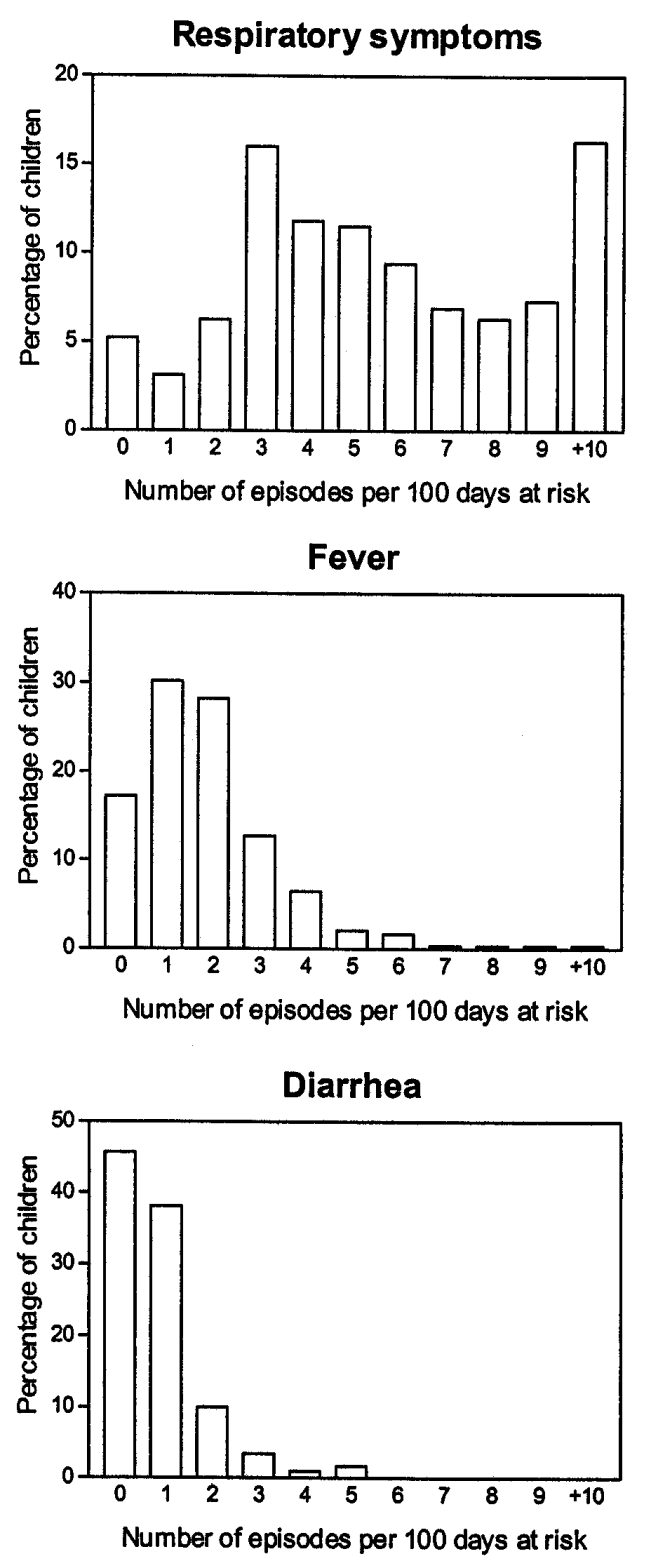
Distribution of number of episodes (respiratory symptoms, reported fever, and diarrhea) per 100 days at risk in 294 children, Sisimiut, Greenland, 1996-1998.

**Figure 2:**
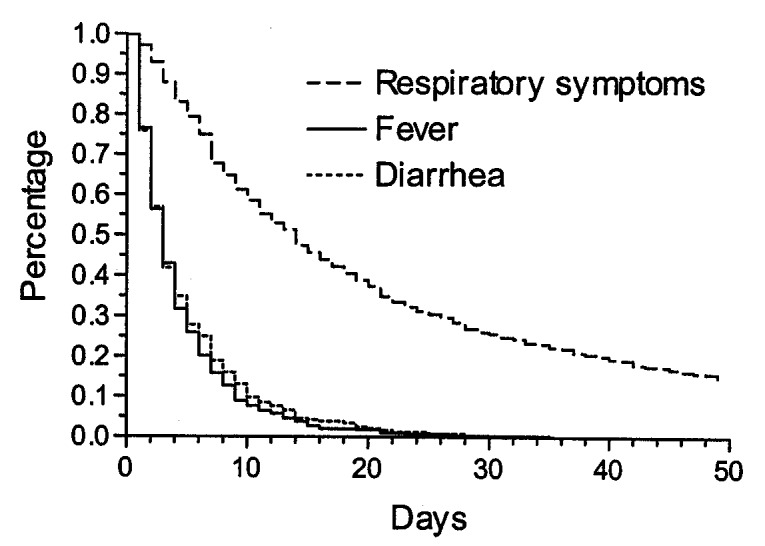
Duration of episodes (respiratory symptoms, reported fever, and diarrhea) in 294 children, Sisimiut, Greenland, 1996-1998.

The prevalence of respiratory symptoms, fever, and diarrhea was highest in the age group 6–11 months. Respiratory symptoms and diarrhea, but not fever, were reported more often for boys than for girls (Table 2). The corresponding incidence of respiratory symptoms was almost 3 times higher than the incidence of fever and 6 times higher than that of diarrhea. The incidence of respiratory symptoms and fever was similar for boys and girls, but boys had substantially more episodes of diarrhea than girls. For all three illnesses, a steep rise in incidence was seen from the age <5 months to 6–11 months, followed by decreasing incidence up to 2 years of age (Table 3). For respiratory symptoms, 5% of the children had no episodes, 37% had 1 to 4 episodes, 41% had 5 to 9 episodes, and 16% had >10 episodes. The median number was 5 (IQR 3 to 8 episodes). For fever and diarrhea, the numbers were much smaller, as 17% and 46%, respectively, of the children had no episodes. The median number of episodes of fever and diarrhea was two and one, respectively (Figure 1). The median duration of respiratory symptom episodes was 14 days (IQR 7 to 33 days) and 3 days for both fever (IQR 2 to 6 days) and diarrhea episodes (IQR 2 to 7 days), respectively (Figure 2).

The most frequently reported symptom was nasal secretion, reported in 26,254 (83%) of days with symptoms, either as the only symptom (37%) or in combination with symptoms of URI (46%). Symptoms of LRI (fast or difficult breathing and chest indrawing) were found in 1.8% of days with respiratory symptoms. Ear discharge indicative of acute or chronic otitis media was present in 4.9% of days of observation.

### URI and LRI Episodes

**Figure 3:**
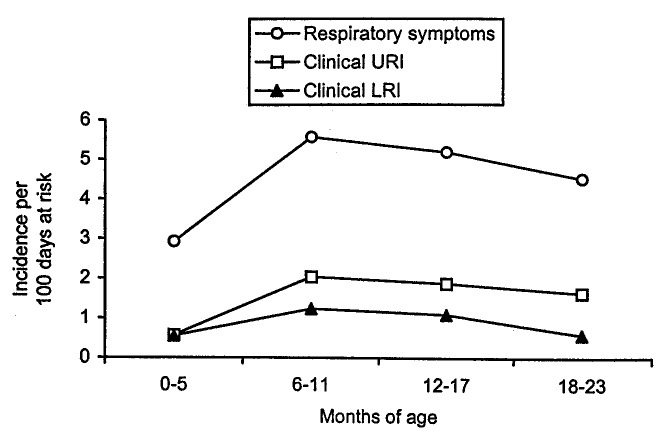
Age-specific incidence of episodes of respiratory symptoms and episodes clinically characterized as upper (URI) or lower respiratory tract infections (LRI) per 100 days at risk in 288 children, Sisimiut, Greenland, 1996-1998.

Six of the 294 participating children did not have 7 consecutive days free of respiratory symptoms before any episode of ARI, leaving 288 children at risk of clinically characterized episodes of acute respiratory infections. Of the 1,547 episodes of respiratory symptoms, 918 were classified as episodes of URI (527), LRI (292), or clear nasal discharge (99) (Table 4). The incidence of LRI was higher in boys than in girls (p<0.001), but no difference in the sex of the child was observed for URI (p=0.329) (Figure 3).

The remaining 629 (41%) of the 1,547 episodes of respiratory symptoms could not be clinically characterized. This group included episodes ending before visit (129), episodes without abnormal clinical signs at examination (301), and episodes for which clinical examinations could not be conducted (199). These episodes were distributed similarly with respect to sex and age

**Figure 4:**
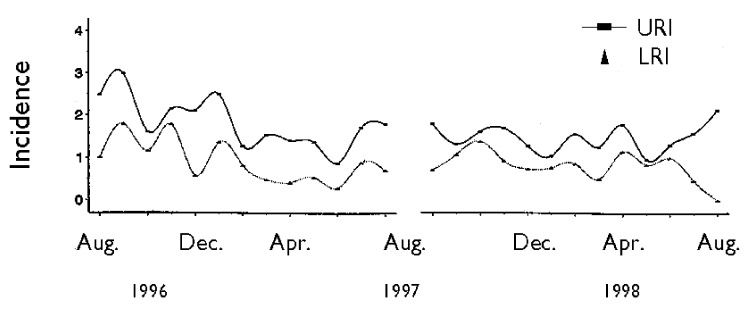
Incidence of clinical episodes of upper respiratory tract infections (URI) and lower respiratory tract infections (LRI) by calendar month in 288 children, Sisimiut, Greenland, 1996-1998.

No seasonal pattern of the incidence of the overall ARI, URI, or LRI was observed (Figure 4). In addition, no seasonal variation was observed for the severe episodes of ARI (e.g., those that required medical attention).

### Severity of Clinical Episodes

Median duration of URI episodes was 14 days (IQR 7–25 days) and of LRI episodes 19 days (IQR 9–39 days). Activity restriction characterized 65% of clinical episodes (58% URI, 75% LRI); 40% of episodes (32% URI, 56% LRI) resulted in outpatient hospital visits. Only one URI and eight LRI episodes caused hospital admittance. No children in the study died from ARI.

## Discussion

The relatively small size of Sisimiut allowed us to invite all children in this community to participate, in contrast to other studies, in which subgroups of children were chosen as study populations (17–22). The participation rate of 87.6% in this study was high compared with rates of 74% and 78% reported in other community studies in the United States (19, 20). We had a low dropout rate (17.7%) and the main cause of lack of follow-up (71.2%) was migration from Sisimiut. We do not believe that our results were biased by selective dropout.

Although living standards in Sisimiut are slightly higher than in Greenland as a whole (23, 24), enough similarities exist within towns and settlements in Greenland that the estimates of incidence and prevalence can be considered representative of the population as a whole.

We found that respiratory symptoms were reported in 41.6% of days of observation and that the incidence of respiratory symptom episodes was 4.7 episodes per 100 days at risk (32.6 episodes/100 weeks at risk). For episodes that were clinically characterized, the incidence of URI and LRI combined was 2.5 episodes per 100 days at risk (17.3 episodes/100 weeks at risk). Because health service in Sisimiut is free and easily accessible, we believe that the episodes not diagnosed through the clinic were not severe.

The results we present are high, compared with those in both developing and industrialized countries. In the BOSTID community studies of children <5 years of age from Kenya, Nigeria, Papua New Guinea, the Philippines, Thailand, Colombia, Uruguay, and Guatemala, prevalence of ARI was reported within the range of 21.7% to 40.1%, and incidence of ARI in the range of 12.7 to 27.5 episodes per 100 child weeks at risk (13). In community studies from Tecumseh, Michigan, and Seattle, Washington, incidence of ARI ranged from 8.6 to 11.7 episodes per 100 child weeks (children <1 year of age) (25,26). In addition, duration of episodes in our study (median 14 days) tended to be relatively long. In the BOSTID and Tecumseh studies, the median episodes lasted 1 to 2 weeks (13) (except for one study, which reported a median duration of 5 weeks [25]).

Some differences in design between our study and others may have affected our comparison. To determine an episode, we used the exact days on which parents reported symptoms. Other studies have used whole weeks as units of time, which may overestimate the duration of episodes and underestimate time at risk. We included nasal secretion as a respiratory symptom, while other studies omitted clear nasal secretion from case definitions. Because we found that nasal secretion was reported for 85% of days with symptoms, including this symptom increased the prevalence and incidence in our results. Similar increased prevalence and incidence have been found in studies that also included mild nasal discharge as part of the case definitions (27, 28). Although we made a conservative estimate of ARI by excluding clear nasal discharge from clinical definitions and including only clinically verified episodes of URI and LRI, we still found a high incidence of 17.3 episodes per 100 child weeks at risk in Sisimiut. In addition, our study population consisted of children <2 years of age. As the incidence of ARI depends on age, the highest incidence seen in children in this age group, the focus on age group might distort the comparison with the BOSTID studies. Nevertheless, compared with two BOSTID studies that specifically studied children <3 years of age, our estimate of 17.3 episodes per 100 child weeks at risk for ARI was higher than the estimates found in these studies (13.2 and 15.4 episodes/100 child weeks at risk) (22,29).

Given that rhinitis symptoms were reported at a high rate, the episodes could be expected to be less severe than in other studies; accordingly, we observed no fatal episodes of ARI. We found that 65% of clinically verified episodes caused activity restriction, and 40% prompted contact with the health center (LRI episodes being more severe than URI episodes). While not completely comparable because of differences in definitions, the severity of episodes appeared to be within the same range as that observed in Tecumseh, where 33% of all respiratory illnesses caused activity restriction (in all age groups) and 47% caused physician visits (children <1 year) (19,25).

We found a significantly increased risk of LRI in boys compared with girls, which is similar to findings of other studies (13,30), although the increased risk for boys in our study was higher than that observed in the BOSTID community studies (13). In contrast, there was no difference in sex of the child with respect to URI, which corresponds with results of Greenlandic studies showing no difference between boys and girls for otitis media and common cold (13-33)Similarly, our finding of the highest risk of both URI and LRI for the 6- to 11- month age group followed by a decline in older age groups is in agreement with results of other studies (13,25). Possible mechanisms for this may include cessation of breast-feeding, degradation of maternal antibodies passively transferred from birth, and attendance at child-care centers.

As some of the episodes were very long, these symptoms could reflect allergy rather than infection. However, episodes characterized by clear nasal secretion as a possible sign of allergic rhinitis were few (only 99 [6.4%] of 1,547 episodes reported), and these episodes were excluded from clinical definitions of ARI. We have recently shown that atopy, defined as elevated specific immunoglobulin E in serum, is half as prevalent in schoolchildren of Sisimiut as in Danish schoolchildren of the same age (34). We are confident that our findings represent infections rather than allergies.

Surprisingly, we found no clear seasonal variation in the incidence of respiratory symptom episodes, clinically verified episodes of URI or LRI, and episodes prompting contact with the health center. Based on hospital contacts and drug prescriptions, the highest incidence of ARI in Greenland has been described in July and in the winter (3,35,36), although other studies failed to demonstrate any seasonal pattern (37). In Greenland, no routine data are available from hospital admissions or routine surveillance for respiratory tract infections or respiratory pathogens to elucidate these findings further. In Alaska, seasonal trends in the incidence of LRI and invasive pneumococcal disease have been described, with highest incidence in the spring and lowest in the fall (4,38); another study found the highest rate of hospitalizations from respiratory syncytial virus in December and lowest in March (39). While seasonal variation may correlate with weather conditions such as low temperature, humidity, and precipitation, proving a causal role of these factors is difficult (30). Instead, causes may be related to crowding in the home correlated with weather conditions (30). While marked variation in monthly average temperature is seen in Sisimiut, ranging from 6.3°C to -14°C in July and March, respectively, relative outdoor humidity varies little (80%–87%). Greenlandic children spend much time outside all year round, even in winter. In child-care centers, the children sleep outside in baby carriages all year unless the temperature drops below -15°C. The lack of seasonal variation in ARI could therefore reflect a pattern of little seasonal variation in indoor stay for children of this age but could also reflect different and opposing patterns of various infectious agents. Studies examining possible seasonality of specific pathogens (e.g., respiratory syncytial virus or Haemophilus influenzae) are warranted.

Although our study focused on acute respiratory tract infections, we also collected data on diarrheal diseases and episodes of fever without other prominent symptoms. These data show that the high illness rate in Sisimiut is specifically caused by ARI and not other infections in childhood, in contrast to data from many developing countries, where young children have high incidences of different kinds of infections. This observation corroborates the point that Sisimiut should be regarded as a modern Greenlandic society with a high incidence of respiratory tract infections and not a developing country setting with high rates of poverty-related diseases, such as diarrhea and malnutrition.

This first population-based community study of ARI in Inuit children <2 years of age based on active surveillance showed a high occurrence of the disease overall. A total of 41.6% of days were spent with symptoms of respiratory tract infections, and the incidence of new episodes of ARI was 2.5 per 100 days at risk. Of all episodes, 65% caused activity restriction, and 40% caused contact with the health center. The prevalence of this disease calls for intervention programs, and further studies are in progress to elucidate risk factors that may allow for specific interventions.

**Table 1.** Study population, Sisimiut, Greenland, 1996–1998

**Table:** 

Characteristics	Participants^a^n (%)	Nonparticipants^b^n (%)	p value
Sex			0.933^c^
Boys	145 (49.3)	22 (50.0)	
Girls	149 (50.7)	22 (50.0)	
Place of birth			0.05^d^
Sisimiut	267 (90.8)	35 (79.5)	
Other Greenlandic towns	20 (6.8)	6 (13.6)	
Denmark	7 (2.4)	3 (6.8)	
Ethnicity^e^			<0.001^d^
Inuit	237 (80.6)	26 (59.1)	
Danish	11 (3.8)	7 (15.9)	
Mixed	30 (10.2)	2 (4.5)	
Unknown	16 (5.4)	9 (20.5)	
Availability for enrollment			<0.001^c^
Available for enrollment before study period^f^	135 (45.9)	7 (15.9)	
Born in Sisimiut in study period	143 (48.6)	31(70.5)	
Moved into Sisimiut in study period	16 (5.4)	6 (13.6)	
Age at first day in monitoring period (mo)^g^			
<2	106 (36.1)		
3–5	42 (14.3)		
6–11	57 (19.4)		
12–17	47 (16.0)		
18–23	42 (14.3)		
Time of illness monitoring (days)			
25% quartile	133		
Median	256		
75% quartile	374		
Range	2–630		
^a^n=294. ^b^Children whose parents refused to participate from the start (n=44). ^c^chi-square test. ^d^Fisher's exact test. ^e^Inuit, both parents born in Greenland. Danish, both parents born in Denmark. Unknown, one or both parents place of birth unknown. ^f^April 1, 1996–June 1, 1998. ^g^Monitoring period July 30 1996–August 13, 1998. Median age 5.8 months.			

**Table 2. **Prevalence of respiratory symptoms, reported fever, and diarrhea in 294 children, Sisimiut, Greenland, 1996–1998

**Table:** 

	Days with symptoms	Days observed (with symptom information)	% ill (prevalence)	p value^a^
Respiratory symptoms				
Total	32,018	76,914	41.6	
Sex				<0.001
Boys	16,060	35,795	44.9	
Girls	15,958	41,119	38.8	
Age (mo)				<0.001
<5	3,242	12,110	26.8	
6–11	9,331	20,926	44.6	
12–17	9,898	22,154	44.7	
18–23	9,547	21,724	43.9	
Fever, reported				
Total	3,763	76,524	4.9	
Sex				0.96
Boys	1,748	35,520	4.9	
Girls	2,015	41,004	4.9	
Age (mo)				<0.001
<5	366	12,101	3.0	
6–11	1,306	20,778	6.2	
12–17	1,213	22,064	5.5	
18–23	878	21,581	4.1	
Diarrhea, reported				
Total	2,017	76,541	2.6	
Sex				<0.001
Boys	1,037	35,526	2.9	
Girls	980	41,015	2.4	
Age (mo)				<0.001
<5	194	12,081	1.6	
6–11	708	20,792	3.4	
12–17	629	22,066	2.9	
18–23	486	21,602	2.2	
^a^Differences in prevalence with respect to sex and age were tested by assuming a binominal distribution.				

**Table 3.** Incidence of episodes of respiratory symptoms, reported fever, and diarrhea in 294 children, Sisimiut, Greenland, 1996–1998

**Table:** 

	No. of new episodes	Days at risk	Incidence/100 days at risk	95% CI^a^	p value^b^
Respiratory symptoms					
Total	1,547	33,228	4.66	4.43, 4.89	
Sex					0.625
Boys	685	14,508	4.72	4.38, 5.09	
Girls	862	18,720	4.60	4.31, 4.92	
Age (mo)					<0.001
<5	201	6,870	2.93	2.55, 3.36	
6–11	471	8,439	5.58	5.10, 6.11	
12–17	462	8,838	5.23	4.77, 5.73	
18–23	413	9,081	4.55	4.13, 5.01	
Calendar periods^c^					0.081
Jan–Mar	393	8,957	4.39	3.97, 4.84	
Apr–Jun	391	8,954	4.37	3.95, 4.82	
Jul–Sep	403	7,944	5.07	4.60, 5.59	
Oct–Dec	360	7,373	4.88	4.40, 5.41	
Fever, reported					
Total	1,106	63,584	1.74	1.64, 1.85	
Sex					0.538
Boys	503	29,505	1.70	1.56, 1.86	
Girls	603	34,079	1.77	1.63, 1.92	
Age (mo)					<0.001
<5	112	10,059	1.11	0.93, 1.34	
6–11	371	16,689	2.22	2.01, 2.46	
12–17	358	18,217	1.97	1.77, 2.18	
18–23	265	18,619	1.42	1.26, 1.61	
Calendar periods^c^					<0.001
Jan–Mar	309	16,609	1.86	1.66, 2.08	
Apr–Jun	230	16,921	1.36	1.19, 1.55	
Jul–Sep	314	15,326	2.05	1.83, 2.29	
Oct–Dec	253	14,728	1.72	1.52, 1.94	
Diarrhea, reported					
Total	523	69,255	0.76	0.69, 0.82	
Sex					0.016
Boys	268	31,833	0.84	0.75, 0.95	
Girls	255	37,422	0.68	0.60, 0.77	
Age (mo)					<0.001
<5	48	10,623	0.45	0.34, 0.60	
6–11	158	18,705	0.84	0.72, 0.99	
12–17	179	19,981	0.90	0.77, 1.04	
18–23	138	19,946	0.69	0.59, 0.82	
Calendar periods^c^					<0.001
Jan–Mar	215	17,423	1.23	1.08, 1.41	
Apr–Jun	50	18,516	0.27	0.20, 0.36	
Jul–Sep	101	17,419	0.58	0.48, 0.70	
Oct–Dec	157	15,897	0.99	0.84, 1.15	
^a^CI, confidence interval. ^b^Likelihood-ratio test. ^c^Months accumulated in monitoring period (July 30, 1996–August 13, 1998).					

**Table 4.** Number and characteristics of clinically characterized episodes of acute respiratory infections in 288 children, Sisimiut, Greenland, 1996–1998^a^

**Table:** 

	No. of new episodes	Days at risk	Incidence/100 days at risk	95% CI^b^	p value^c^
URI					
Total	527	33,228	1.59	1.46, 1.73	
Sex					0.329
Boys	219	14,508	1.51	1.32, 1.72	
Girls	308	18,720	1.65	1.47, 1.84	
Age (mo)					<0.001
<5	39	6,870	0.57	0.41, 0.78	
6–11	172	8,439	2.04	1.76, 2.37	
12–17	167	8,838	1.89	1.62, 2.20	
18–23	149	9,081	1.64	0.41, 0.78	
LRI					
Total	292	33,228	0.88	0.78, 0.99	
Sex					<0.001
Boys	159	14,508	1.10	0.94, 1.28	
Girls	133	18,720	0.71	0.60, 0.84	
Age (mo)					<0.001
<5	38	6,870	0.55	0.40, 0.76	
6–11	104	8,439	1.23	1.02, 1.49	
12–17	97	8,838	1.10	0.90, 1.34	
18–23	53	9,081	0.58	0.45, 0.76	
Clear nasal discharge^d^					
Total	99	33,228	0.30	0.24, 0.36	
Sex					0.021
Boys	32	14,508	0.22	0.16, 0.31	
Girls	67	18,720	0.36	0.28, 0.45	
Age (mo)					0.002
<5	7	6,870	0.10	0.05, 0.21	
6–11	32	8,439	0.38	0.27, 0.54	
12–17	33	8,838	0.37	0.27, 0.53	
18–23	27	9,081	0.30	0.20, 0.43	
^a^Six of the 294 participating children did not have 7 consecutive days free of respiratory symptoms before any episode of ARI, leaving 288 children at risk of clinically characterized episodes of acute respiratory infections. ^b^Abbreviations used: CI, confidence interval; URI, upper respiratory tract infections; LRI, lower respiratory tract infections; ARI, acute respiratory infections. ^c^Likelihood-ratio. ^d^Based on the medcal students' clinical examinations only, as doctors at the community health center did not discriminate between clear and purulent nasal secretions.					
